# Expression of Concern: Emerging roles of the long non-coding RNA 01296/microRNA-143-3p/MSI2 axis in development of thyroid cancer

**DOI:** 10.1042/BSR-2018-2376_EOC

**Published:** 2022-09-26

**Keywords:** AKT/STAT3 signaling pathway, Competing endogenous RNA, Long non-coding RNA 01296, microRNA-143-3p, Musashi 2, Thyroid cancer

The Editorial Office has been made aware of potential issues surrounding the scientific validity of this paper, hence has issued an Expression of Concern to notify readers whilst the Editorial Office investigates. An apparent duplication has been identified between the si-NC and the miR-143-3p mimic + pcDNA-MSI2 panels of Figure 7H.

